# State of the art of ICD programming: Lessons learned and future directions

**DOI:** 10.1007/s12471-014-0582-4

**Published:** 2014-07-30

**Authors:** M. H. Mastenbroek, S. S. Pedersen, H. Versteeg, P. A. Doevendans, M. Meine

**Affiliations:** 1 Cardiology, Department of Heart and Lung, University Medical Center, Heidelberglaan 100, PO Box 85500, 3584 CX Utrecht, the Netherlands; 2 CoRPS - Center of Research on Psychology in Somatic diseases, Tilburg University, Tilburg, the Netherlands; 3 Department of Cardiology, Erasmus Medical Center Rotterdam, Rotterdam, the Netherlands; 4 Department of Cardiology, Odense University Hospital, Odense, Denmark; 5 Department of Psychology, University of Southern Denmark, Odense, Denmark

**Keywords:** ENHANCED-ICD, Implantable cardioverter defibrillator, ICD programming, Quality of life

## Abstract

The lifesaving benefits of implantable cardioverter defibrillator (ICD) therapy are more and more weighted against possible harm (e.g. unnecessary device therapy, procedural complications, device malfunction etc.) which might have adverse effects on patients’ perceived health status and quality of life. Hence, there has been an increasing interest in the optimisation of ICD programming to prevent inappropriate and appropriate but unnecessary device therapy. The purpose of the current report is to give an overview of research into the optimisation of ICD programming and present the design of the on-going ENHANCED-ICD study. The ENHANCED-ICD study is a prospective, safety monitoring study enrolling 60 primary and secondary prophylactic ICD patients at the University Medical Center Utrecht. Patients implanted with any type of ICD with SmartShock technology^TM^, and between 18–80 years of age, were eligible to participate. In all patients a prolonged detection of 60/80 intervals was programmed. The primary objective of the study is to investigate whether enhanced programming to further reduce ICD therapies is safe. The secondary objective is to examine the impact of enhanced programming on *(i)* antitachycardia pacing and shocks (both appropriate and inappropriate) and *(ii)* quality of life and distress. The first results of the ENHANCED-ICD study are expected in 2015.

## Introduction

When implantable cardioverter defibrillator (ICD) treatment was initially introduced, ICDs were exclusively implanted in patients with documented sustained ventricular tachycardia (VT) or ventricular fibrillation (VF) to prevent sudden cardiac death (SCD, secondary prevention). Nowadays, the ICD is the first-line treatment for a much broader population of patients, including patients with an anticipated risk for arrhythmic death (primary prevention) [1]. While in the past mortality reduction was the primary goal of ICD therapy, in the last decade this lifesaving benefit is more and more weighted against possible harm in the form of unnecessary device therapy, proarrhythmic potential, procedural complications, infection, device malfunction, and manufacturer recalls, which might have adverse effects on patients’ perceived health status and quality of life [2, 3]. Hence, there has been an increasing interest in the optimisation of ICD programming to prevent inappropriate and unnecessary appropriate device therapy (either antitachycardia pacing (ATP) or shock), as it is associated with heart failure and prognosis, and may lower patient-perceived health status. The purpose of the current report is to give an overview of research into the optimisation of ICD programming and present the design of the *ENHANCED device programming to reduce therapies and improve quality of life in Implantable Cardioverter Defibrillator patients (ENHANCED-ICD) study*, which started in April 2013.

### Prolonged life at the expense of unnecessary ICD therapy

There is no doubt about the efficacy of ICD therapy in prolonging the life of patients who are at risk for SCD [4–6]. Secondary prevention ICD trials (e.g. the Antiarrhythmics Versus Implantable Defibrillators (AVID) trial, the Canadian Implantable Defibrillator Study (CIDS), and the Cardiac Arrest Study Hamburg (CASH)) have shown high numbers of appropriate ATP and shocks in patients during follow-up [7–9], whereas these numbers are lower in patients receiving an ICD for primary prevention [5, 6, 10]. Remarkably, the Multicenter Automated Defibrillator Implantation Trial II (MADIT II) [6] and the Sudden Cardiac Death in Heart Failure Trial (SCD-HeFT) [5], both primary prevention ICD trials, showed that the number of appropriate ICD therapies for VT/VF in the ICD group outnumbered sudden cardiac arrest in the control group (either conventional medical therapy with or without amiodarone) by a factor of 2 to 3 [11]. Thus, ICD therapy was probably delivered for non-life-threatening ventricular tachyarrhythmias (haemodynamically stable or non-sustained VTs).

### Painfree therapy for fast VT

One of the first trials that aimed to reduce appropriate ICD shocks was the Pacing Fast VT Reduces Shock Therapies (PainFREE Rx II) trial, which started in 2001 and randomised ICD patients to either ATP or shock as first therapy for fast VT (FVT) [12]. It was the first prospective randomised trial to demonstrate that ATP was safe and effective compared with shocks for treating FVT. ATP terminated 73 % of FVT episodes (which made up 76 % of all ventricular arrhythmias conventionally programmed to shock) with a very low risk of acceleration and syncope and no difference in mortality. Subsequently, the Avoid Delivering Therapies for Non-Sustained Arrhythmias in ICD Patients III (ADVANCE III) trial showed that in prolonged arrhythmia detection ATP efficacy (with an ATP-during-charge feature) was still as high as 44 % [13].

### Noise – SVT – VT discrimination

The most common triggers for inappropriate shocks are supraventricular tachycardia (SVT), intracardiac oversensing, lead fracture or other extracardiac noise [14, 15]. This knowledge has led to the development of more sophisticated in-device automated detection algorithms to increase ICD specificity without reducing sensitivity when treating patients at risk for SCD. In the PainFREE Rx II trial, over 11 ± 3 months, inappropriate therapies due to misclassification of rapidly conducted SVTs occurred in 15 % of primary and secondary prevention patients and accounted for more than one-third of all therapies and ≈ 40 % of all shocks in both groups [16]. The PainFREE SST study was designed to investigate the ability of new algorithms (SmartShock^™^ Technology) to reduce inappropriate shocks by enrolling up to 2000 primary and secondary prophylactic patients implanted with an ICD or cardiac resynchronisation therapy defibrillator (CRT-D) device. First results showed that this new technology resulted in a low incidence of inappropriate ICD shocks (1.6 and 2.3 % in primary and secondary prevention patients, respectively), while maintaining flexibility in detection rate [17].

### Detection duration and unnecessary ICD therapy

The fact that 34 % of detected FVT episodes in the shock arm of the PainFREE Rx II trial terminated during the 3.3 s (median) of capacitor charging suggested that a longer delay would further reduce unnecessary ATP [12]. It was intuitively assumed that increased duration of tachycardia might increase syncope. However, delaying detection to a number of intervals to detect (NID) of 18 out of 24 beats (18/24) proved safe because arrhythmic syncope (8 of 1837, 0.5 %) did not increase compared with PainFREE Rx (13 of 1248, 2.0 %), which used a NID of 12/16 [18]. The Primary Prevention Parameters Evaluation (PREPARE) study, a prospective nonrandomised cohort-controlled ICD trial which started in 2003, enrolled 700 primary prevention patients and strategically chose VT/VF detection and therapy parameters to reduce shocks and other morbidities. VT/VF was detected for rates ≥182 beats per minute (bpm) that was sustained for at least 30 out of 40 beats. ATP was programmed as first therapy for regular rhythms with rates of 182–250 bpm, and supraventricular tachycardia discriminators were used for rhythms ≤200 bpm. The control group consisted of 689 primary prevention patients from the Comparison of Empiric to Physician-Tailored Programming of Implantable Cardioverter Defibrillators (EMPIRIC) and the Multicenter InSync Implantable Cardioversion Defibrillation Randomized Clinical Evaluation (MIRACLE ICD) trials for whom VT/VF detection and therapy programming were not controlled. The PREPARE study demonstrated that programming a monitor zone without ICD intervention for slower VT episodes, longer arrhythmia-detection duration (i.e. NID 30/40) both in the fast VT ≥182 bpm and VF zone ≥250 bpm, and the use of supraventricular detection discrimination algorithms were associated with reductions in both appropriate and inappropriate shocks in the first year (9 vs. 17 %) and reductions in morbidity index events (0.26 events/patient-year for PREPARE study patients vs. 0.69 for control cohort patients) [19]. In the recently completed PainFREE SST study, which started in 2009, the safety of extending VT/VF interval detection duration (18/24 vs. 30/40 intervals) is assessed. Primary prophylactic patients received a VF NID of 30/40, while secondary prophylactic patients were randomised to a VF NID of either 18/24 or 30/40 [20]. Results from this study are expected soon. The MADIT-RIT (Reduce Inappropriate Therapy), a large-scale, randomised trial which also started in 2009, assessed the impact of high-rate cut-offs and longer delays than standard programming on inappropriate therapy in primary prevention patients receiving an ICD (dual-chamber) or CRT-D. A total of 1500 hundred patients were randomly assigned to one of three programming configurations: *conventional* (VT between 170–199 bpm with a 2.5-s delay and VT/SVT discriminators turned on; VF ≥200 bpm with a 1-s delay before delivery of ATP or shock), *high rate* (VT monitoring between 170–199 bpm; VF ≥200 bpm with a duration of 2.5-s) and *delayed therapy* (VT-1 between 170–199 bpm, with rhythm detection on and a 60-s delay before initiation of therapy; VT-2 ≥ 200 bpm, with rhythm detection on and a 12-s delay before therapy; and VF ≥250 bpm with a 2.5-s delay before initiation of therapy). As compared with the conventional-therapy group, the high-rate and delayed-therapy groups had significantly fewer patients with a first and total occurrence of appropriate or inappropriate therapy [21]. Findings were dominated by reductions in ATP. First occurrences of inappropriate ATP and shocks were most frequent with regular SVT and atrial fibrillation. The fact that also appropriate ATP occurred less often demonstrated that many VT episodes terminated spontaneously and did not need any ICD therapy [21]. Finally, the ADVANCE III trial, a randomised controlled clinical trial which started in 2008, assessed whether increasing the NID is an effective strategy to further reduce appropriate and inappropriate ICD intervention in any type of ICD (single-chamber, dual-chamber, CRT-D), among patients with both primary and secondary ICD indications. ADVANCE III demonstrated that the use of a long detection setting (NID 30/40) in ICDs with the capability of delivering ATP during capacitor charge significantly reduced the rate of appropriate therapies (ATP and shocks) and inappropriate shocks compared with the standard detection setting (NID 18/24) [13]. Mortality and syncope rates did not significantly differ between the groups. A NID of 30/40 also avoided an appropriate shock in 54 % of the sustained episodes with cycle length between 240–320 ms. These results confirmed and reinforced, in a larger population, the main results presented by the MADIT-RIT trial.

### ICD therapy and psychological well-being

Shocks have also been shown to impact adversely on mental well-being and physical functioning [22, 23], although the evidence for an adverse effect of shocks on these outcomes is not consistent, even in the major primary and secondary prevention trials that included quality of life as a secondary endpoint [24]. Moreover, there is increasing evidence that the psychological status and profile of the ICD patient is an important determinant of both the onset of VT [25–27], but also survival [27–30].

### ENHANCED-ICD study

**Fig 1:**
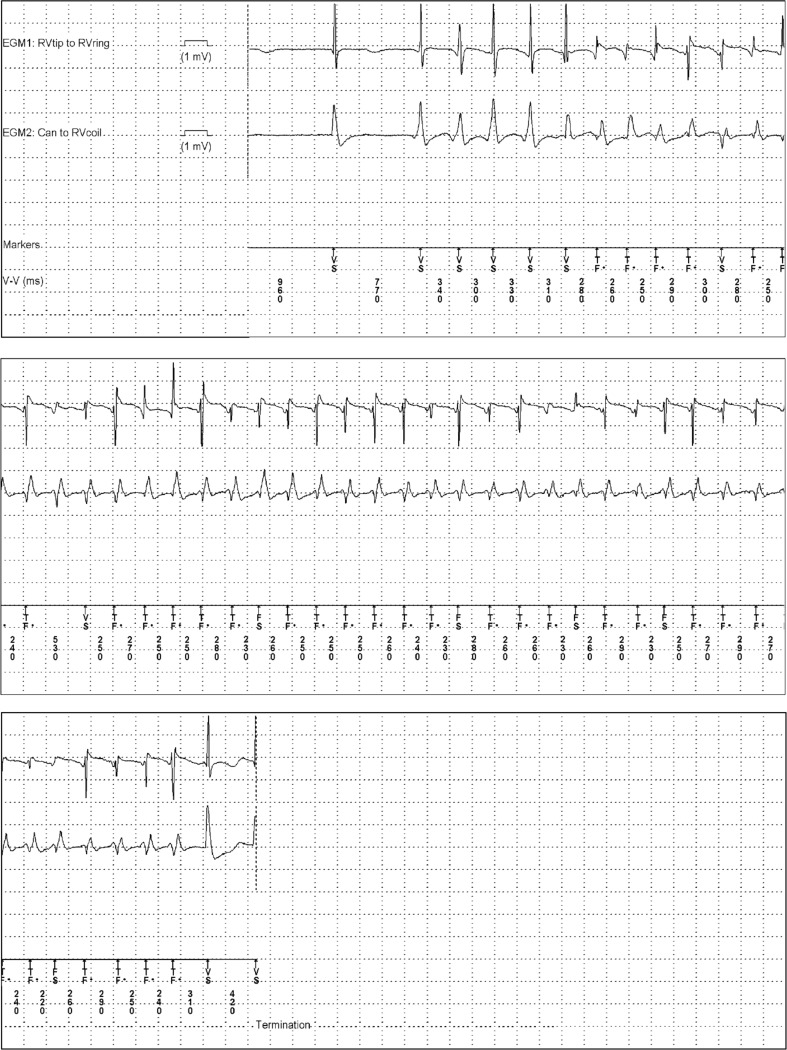
EGM of an ENHANCED-ICD patient whose VT spontaneously terminated after >30 intervals

Despite new detection algorithms approximately 2 % of primary and secondary prophylactic ICD patients receive an inappropriate shock during the first year [17]. The number of appropriate but unnecessary ICD therapies may be even higher. One important tool to reduce both appropriate and inappropriate ICD therapy is to prolong the tachycardia detection duration. To provide an example, a patient with a VT of 200 bpm (cycle length =300 ms) and a NID of 30/40, may already receive ATP after 9 s (30 * 300 ms = 9000 ms). However, ICD therapy might be unnecessary because of spontaneous termination of an episode after 9 s, as illustrated in Fig. 1. In this patient from the on-going ENHANCED-ICD study, unnecessary ATP could be avoided by prolonging the NID to 60/80.

The on-going ENHANCED-ICD study should be seen as an extension of the PREPARE, PainFREE SST and ADVANCE III trials rather than a duplicate, as this study aims to reduce the number of ICD therapies even further (by increasing the NID to 60/80), while also including the patient perspective by using questionnaires on patient quality of life in the broadest sense due to inclusion of anxiety and depression and other psychological risk markers that have been shown to influence not only time to onset of tachyarrhythmias but also mortality in ICD patients. The ENHANCED-ICD study is a prospective, single-arm safety monitoring study which was designed together with Tilburg University and implemented in the University Medical Center Utrecht (UMCU), the Netherlands. Patients implanted with a CE-approved and market-released ICD (single-chamber or dual-chamber) or CRT-D device with SST, between 18–80 years of age, and eligible for primary or secondary prophylactic ICD or CRT-D therapy according to the current guidelines were eligible to participate. Patients on the waiting list for heart transplantation, with a history of psychiatric illness other than affective/anxiety disorders, or unable to complete the questionnaires due to cognitive impairments, or insufficient knowledge of the Dutch language were excluded. The primary objective of the study is to investigate whether enhanced programming (*VT monitoring zone* >166/min; *FVT zone* ≥ 182/min via VF NID 60/80 with 3x ATP and if unsuccessful followed by shock; *VF* ≥ 250/min with 1x ATP during charging and if unsuccessful followed by shock) to reduce therapy is safe for patients with a primary or secondary ICD indication. The secondary objective is to examine the impact of enhanced programming on *(i)* ATP and shocks (both appropriate and inappropriate) and *(ii)* quality of life and distress. Based on the sample size calculation, we needed to include at least 60 patients in our study to be able to decide on whether enhanced programming is safe or not. Enhanced programming is considered safe if the number of arrhythmic syncopes and other intervention-related safety events (either hospitalisation, death or other serious adverse event due to Enhanced programming) does not exceed the safety threshold. To closely monitor safety, an established, sequential safety monitoring model is used [31]. The study will be prematurely closed if the number of intervention-related safety events exceeds the safety threshold. Patient inclusion started on 15 April 2013 and the last patient was included on 20 December 2013. The device follow-up assessments take place at 2, 6 and 12 months post implantation (standard procedure), and every 6 months afterwards until the last included patient has completed the 12-month follow-up (end of study). In addition, all patients were connected to remote patient monitoring. At baseline (pre-implantation), 3, 6 and 12 months post-implantation patients fill in a questionnaire measuring quality of life and distress.

In conclusion, considerable efforts have been made to reduce inappropriate and unnecessary device therapy (ATP and shock) in ICD patients. As recently stated by Helmut Klein (adjunct professor of Medicine, University of Rochester Medical Center, USA), *‘there is a paradigm shift of ICD programming to less fast and aggressive arrhythmia termination using prolonged detection, delayed intervention and no therapy delivery for slower and stable VT events, allowing them to terminate spontaneously*’ [32]. The ENHANCED-ICD study will examine if further reductions in inappropriate and unnecessary ICD therapy are possible and look at the associated impact on patient well-being, with results expected in 2015.

## References

[1] Ezekowitz JA, Armstrong PW, McAlister FA (2003). Implantable cardioverter defibrillators in primary and secondary prevention: a systematic review of randomized, controlled trials. Ann Intern Med.

[2] Kraaier K, Starrenburg AH, Verheggen RM, van der Palen J, Scholten MF (2013). Incidence and predictors of phantom shocks in implantable cardioverter defibrillator recipients. Neth Heart J.

[3] Wijers SC, van der Kolk BY, Tuinenburg AE, Doevendans PA, Vos MA, Meine M (2013). Implementation of guidelines for implantable cardioverter-defibrillator therapy in clinical practice: which patients do benefit?. Neth Heart J.

[4] A comparison of antiarrhythmic-drug therapy with implantable defibrillators in patients resuscitated from near-fatal ventricular arrhythmias. The Antiarrhythmics versus Implantable Defibrillators (AVID) Investigators. N Engl J Med. 1997;337:1576–83. 10.1056/NEJM199711273372202 9411221

[5] Bardy GH, Lee KL, Mark DB, Poole JE, Packer DL, Boineau R (2005). Amiodarone or an implantable cardioverter-defibrillator for congestive heart failure. N Engl J Med.

[6] Moss AJ, Zareba W, Hall WJ, Klein H, Wilber DJ, Cannom DS (2002). Prophylactic implantation of a defibrillator in patients with myocardial infarction and reduced ejection fraction. N Engl J Med.

[7] Connolly SJ, Gent M, Roberts RS, Dorian P, Green MS, Klein GJ (1993). Canadian implantable defibrillator study (CIDS): study design and organization CIDS Co-Investigators. Am J Cardiol.

[8] Klein RC, Raitt MH, Wilkoff BL, Beckman KJ, Coromilas J, Wyse DG (2003). Analysis of implantable cardioverter defibrillator therapy in the antiarrhythmics versus implantable defibrillators (AVID) trial. J Cardiovasc Electrophysiol.

[9] Siebels J, Cappato R, Ruppel R, Schneider MA, Kuck KH (1993). Preliminary results of the cardiac arrest study hamburg (CASH) CASH Investigators. Am J Cardiol.

[10] Multicenter automatic defibrillator implantation trial (MADIT): design and clinical protocol. MADIT Executive Committee. Pacing Clin Electrophysiol. 1991;14:920–7. 10.1111/j.1540-8159.1991.tb04136.x 1712462

[11] Germano JJ, Reynolds M, Essebag V, Josephson ME (2006). Frequency and causes of implantable cardioverter-defibrillator therapies: is device therapy proarrhythmic?. Am J Cardiol.

[12] Wathen MS, DeGroot PJ, Sweeney MO (2004). Prospective randomized multicenter trial of empirical antitachycardia pacing versus shocks for spontaneous rapid ventricular tachycardia in patients with implantable cardioverter-defibrillators: pacing fast ventricular tachycardia reduces shock therapies (PainFREE Rx II) trial results. Circulation.

[13] Gasparini M, Proclemer A, Klersy C (2013). Effect of long-detection interval vs standard-detection interval for implantable cardioverter-defibrillators on antitachycardia pacing and shock delivery: the ADVANCE III randomized clinical trial. JAMA.

[14] Poole JE, Johnson GW, Hellkamp AS (2008). Prognostic importance of defibrillator shocks in patients with heart failure. N Engl J Med.

[15] Koneru JN, Swerdlow CD, Wood MA, Ellenbogen KA (2011). Minimizing inappropriate or “unnecessary” implantable cardioverter-defibrillator shocks: appropriate programming. Circ Arrhythm Electrophysiol.

[16] Sweeney MO, Wathen MS, Volosin K (2005). Appropriate and inappropriate ventricular therapies, quality of life, and mortality among primary and secondary prevention implantable cardioverter defibrillator patients: results from the pacing fast VT REduces Shock ThErapies (PainFREE Rx II) trial. Circulation.

[17] Schloss E, Auricchio A, Kurita T, et al. PainFree SST trial primary results: low shock rates in patients with dual and triple chamber ICDs using novel detection algorithms. Europace. 2013;15:ii116-ii7.

[18] Wathen MS, Sweeney MO, DeGroot PJ (2001). Shock reduction using antitachycardia pacing for spontaneous rapid ventricular tachycardia in patients with coronary artery disease. Circulation.

[19] Wilkoff BL, Williamson BD, Stern RS (2008). Strategic programming of detection and therapy parameters in implantable cardioverter-defibrillators reduces shocks in primary prevention patients: results from the PREPARE (primary prevention parameters evaluation) study. J Am Coll Cardiol.

[20] Auricchio A, Meijer A, Kurita T (2011). Safety, efficacy, and performance of new discrimination algorithms to reduce inappropriate and unnecessary shocks: the PainFree SST clinical study design. Europace.

[21] Moss AJ, Schuger C, Beck CA (2012). Reduction in inappropriate therapy and mortality through ICD programming. N Engl J Med.

[22] Schron EB, Exner DV, Yao Q (2002). Quality of life in the antiarrhythmics versus implantable defibrillators trial: impact of therapy and influence of adverse symptoms and defibrillator shocks. Circulation.

[23] Keren A, Sears SF, Nery P (2011). Psychological adjustment in ICD patients living with advisory fidelis leads. J Cardiovasc Electrophysiol.

[24] Pedersen SS, Van Den Broek KC, Van Den Berg M, Theuns DA (2010). Shock as a determinant of poor patient-centered outcomes in implantable cardioverter defibrillator patients: is there more to it than meets the eye?. Pacing Clin Electrophysiol.

[25] Van den Broek KC, Nyklicek I, van der Voort PH, Alings M, Meijer A, Denollet J (2009). Risk of ventricular arrhythmia after implantable defibrillator treatment in anxious type D patients. J Am Coll Cardiol.

[26] Whang W, Albert CM, Sears SF (2005). Depression as a predictor for appropriate shocks among patients with implantable cardioverter-defibrillators: results from the Triggers of Ventricular Arrhythmias (TOVA) study. J Am Coll Cardiol.

[27] Habibovic M, Pedersen SS, van den Broek KC (2013). Anxiety and risk of ventricular arrhythmias or mortality in patients with an implantable cardioverter defibrillator. Psychosom Med.

[28] Ladwig KH, Baumert J, Marten-Mittag B, Kolb C, Zrenner B, Schmitt C (2008). Posttraumatic stress symptoms and predicted mortality in patients with implantable cardioverter-defibrillators: results from the prospective living with an implanted cardioverter-defibrillator study. Arch Gen Psychiatry.

[29] Mastenbroek MH, Versteeg H, Jordaens L, Theuns DA, Pedersen SS (2014). Ventricular tachyarrhythmias and mortality in patients with an implantable cardioverter defibrillator: impact of depression in the MIDAS cohort. Psychosom Med.

[30] Pedersen SS, van den Broek KC, Erdman RA, Jordaens L, Theuns DA (2010). Pre-implantation implantable cardioverter defibrillator concerns and type D personality increase the risk of mortality in patients with an implantable cardioverter defibrillator. Europace.

[31] Whitehead J (1997). The Design and Analysis of Sequential Clinical Trials. Revised Second Edition.

[32] Klein H. The challenge of optimal ICD programming. 2014; Available from: http://www.cxvascular.com/crn-features/cardiac-rhythm-news---features/the-challenge-of-optimal-icd-programming

